# Integrated plasticity at inhibitory and excitatory synapses in the cerebellar circuit

**DOI:** 10.3389/fncel.2015.00169

**Published:** 2015-05-05

**Authors:** Lisa Mapelli, Martina Pagani, Jesus A. Garrido, Egidio D’Angelo

**Affiliations:** ^1^Department of Brain and Behavioral Sciences, University of PaviaPavia, Italy; ^2^Museo Storico Della Fisica e Centro Studi e Ricerche Enrico FermiRome, Italy; ^3^Institute of Pharmacology and Toxicology, University of ZurichZurich, Switzerland; ^4^Brain Connectivity Center, C. Mondino National Neurological InstitutePavia, Italy; ^5^Department of Computer Architecture and Technology, University of GranadaGranada, Spain

**Keywords:** cerebellum, inhibitory synapse, excitatory synapse, LTP, LTD

## Abstract

The way long-term potentiation (LTP) and depression (LTD) are integrated within the different synapses of brain neuronal circuits is poorly understood. In order to progress beyond the identification of specific molecular mechanisms, a system in which multiple forms of plasticity can be correlated with large-scale neural processing is required. In this paper we take as an example the cerebellar network, in which extensive investigations have revealed LTP and LTD at several excitatory and inhibitory synapses. Cerebellar LTP and LTD occur in all three main cerebellar subcircuits (granular layer, molecular layer, deep cerebellar nuclei) and correspondingly regulate the function of their three main neurons: granule cells (GrCs), Purkinje cells (PCs) and deep cerebellar nuclear (DCN) cells. All these neurons, in addition to be excited, are reached by feed-forward and feed-back inhibitory connections, in which LTP and LTD may either operate synergistically or homeostatically in order to control information flow through the circuit. Although the investigation of individual synaptic plasticities *in vitro* is essential to prove their existence and mechanisms, it is insufficient to generate a coherent view of their impact on network functioning *in vivo*. Recent computational models and cell-specific genetic mutations in mice are shedding light on how plasticity at multiple excitatory and inhibitory synapses might regulate neuronal activities in the cerebellar circuit and contribute to learning and memory and behavioral control.

## Introduction

Various persistent modifications in neuronal and synaptic functioning provide the biological basis of learning and memory in neuronal circuits and, among these, long-term synaptic plasticity (Bliss and Collingridge, 1993) and intrinsic neuronal excitability (Linden, 1999; Hansel et al., 2001; Xu and Kang, 2005) are thought to play a primary role. Long-term synaptic plasticity appears in various forms of potentiation (LTP) and depression (LTD). Although different forms and mechanisms of LTP and LTD have been revealed, often along with forms of intrinsic excitability changes occurring in the same neurons, plasticity in inhibitory subcircuits is still poorly understood. Moreover, the way inhibitory and excitatory mechanisms cooperate in determining brain circuit computations remains unclear. What is most critical is to understand how excitatory and inhibitory plasticity impinging on the same neuron regulate its function, and how excitatory and inhibitory plasticity contribute to microcircuit computation as a whole. This lack of knowledge is somewhat surprising if one considers that long-term synaptic plasticity is largely believed to play a key role in regulating neuronal and microcircuit operations.

**Figure 1:**
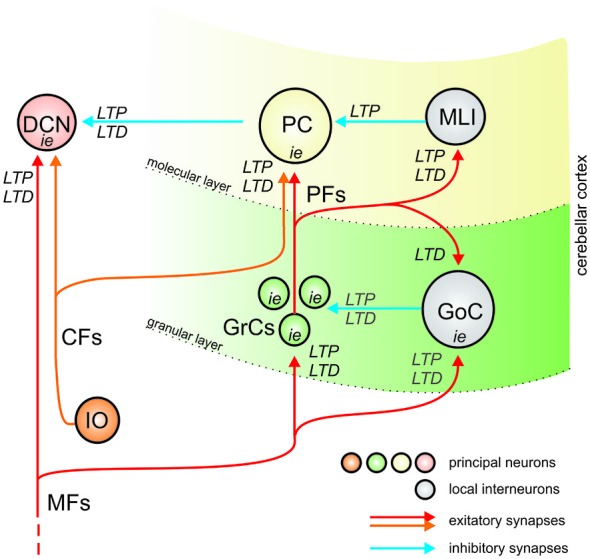
**The organization of plasticity in the cerebellar circuit**. The drawing shows that the cerebellum is made of three main sub-circuits, comprising the granular layer, molecular layer and deep cerebellar nuclear (DCN). The granular layer and molecular layer form the cerebellar cortex. Both the cerebellar cortex and DCN are activated by MFs, and the cerebellar cortex output inhibits the DCN. Therefore, the cerebellar cortex forms a large inhibitory loop for DCN. Inside cerebellar cortex, in turn, MFs activate GrCs which emit PFs activating Purkinje cells (PCs) and local interneurons inhibit the principal neurons (GoCs inhibit GrCs, MLIs inhibit PCs). PCs and DCN cells are also activated by Climbing fibers (CFs). Therefore, a similar feed-forward inhibitory scheme is implemented in all the three cerebellar subcircuits. *LTP*, *LTD* and plasticity of intrinsic excitability (i.e.,) have been either observed or predicted in all subcircuits. The forms of plasticity determined experimentally are reported in *black*, those predicted by computational modeling are reported in *gray*. Excitatory and inhibitory synapses are represented using *red* and *blue* arrows.

In the cerebellum, long-term synaptic plasticity was initially predicted on theoretical grounds to occur only in the form of LTD or LTP (Marr, 1969; Albus, 1971) at the parallel fiber—Purkinje cell (PF-PC) synapse, but now synaptic plasticity is known to be distributed in the granular layer, molecular layer and deep cerebellar nuclear (DCN; Hansel et al., 2001; Gao et al., 2012) involving both excitatory and inhibitory synaptic transmission as well as neuronal intrinsic excitability. These different forms of plasticity eventually impinge on three main neurons, namely GrCs, Purkinje cells (PCs), and DCN cells, which act therefore as nodes integrating excitatory and inhibitory plasticity (Figure 1). Thus, the cerebellum is an ideal system in which the interplay of excitatory and inhibitory plasticity can be investigated. Following their discovery, the possible role of cerebellar plasticities has been hypothesized:

1. Synaptic plasticity in the granular layer may serve to improve spatio-temporal recoding of mossy fiber (MF) inputs into new GrC spike patterns [expansion recoding (D’Angelo and De Zeeuw, 2009)]. Plasticity in the inhibitory Golgi cell (GoC) loop has still to be fully investigated but, based on modeling predictions, it may provide a powerful regulatory mechanism for transmission of appropriate spike trains to PCs.
2. Synaptic plasticity in the molecular layer may serve to store correlated granular layer spike patterns under the teaching signal generated by climbing fibers (CFs) although this latter point is controversial (D’Angelo et al., 2011). This plasticity is in fact composed of multiple mechanisms: different forms of PF-PC LTD and LTP occur together with plasticity in the molecular layer inhibitory interneuron (MLI) network involving GABAergic synapses. For example, PF-PC LTD may occur together with PF-MLI LTP and MLI-PC LTP globally raising PC responses, while PF-PC LTP may occur together with PF-MLI LTD and MLI-PC LTD globally reducing PC responses (Gao et al., 2012).
3. Synaptic plasticity in the DCN may serve to store MF spike patterns (Bagnall and du Lac, 2006; Pugh and Raman, 2006) depending on control signals generated through the cerebellar cortical loop (Figure 1). The inhibitory PCs synapses, which regulate DCN activity (Hansel et al., 2001; Boyden et al., 2004; Gao et al., 2012), develop their own LTP and LTD (Morishita and Sastry, 1996; Aizenman et al., 1998; Ouardouz and Sastry, 2000). Recent works (Medina and Mauk, 1999, 2000; Masuda and Amari, 2008) have suggested the importance for MF-DCN and PC-DCN plasticity in controlling cerebellar learning in eye-blink conditioning and vestibulo-ocular reflex (VOR).
4. Long-term changes in intrinsic excitability in GrCs, PCs and DCN cells could further contribute to change the global activity level in these neurons contributing to homeostasis and plasticity (e.g., see Schweighofer et al., 2001).

In this review we evaluate the integrated impact of plasticity at inhibitory and excitatory synapses along with long-term changes in intrinsic excitability in the cerebellar circuit and highlight their implications for cerebellar computation.

## Long-Term Synaptic Plasticity and Learning in the Cerebellar Circuit

The cerebellum is classically associated with motor control, and learning is thought to subserve the role of calibrating synaptic weights for appropriate response gain regulation and timing. The cerebellum is thought to act through cerebro-cerebellar loops involving the motor cortices (Eccles et al., 1972; Ito, 1972). The critical role in executing precise movements becomes evident when studying patients with cerebellar malfunctioning and diseases, who manifest a sensori-motor syndrome called *ataxia*. Nevertheless, in the last decade a growing body of evidence supported the cerebellar involvement in non-motor, cognitive and emotional functions (Schmahmann, 2010; Schraa-Tam et al., 2012; Voogd, 2012). It is likely that the cerebro-cerebellar loops involved in motor control represent also a model of how the cerebellum takes part in higher functions through reciprocal connections with non-motor brain areas (D’Angelo and Casali, 2012).

The cerebellum controls movements on the millisecond time scale. The motor commands descending from upstream brain areas are relayed to the cerebellum through the pontine nuclei. Once elaborated in the cerebellar circuits, these signals are sent back to the motor cortex through the thalamus to trigger motor acts with appropriate timing (Timmann et al., 1999). The ability to elaborate temporal information on the millisecond time scale led to consider the cerebellum as a *timing* machine (Eccles et al., 1967; Eccles, 1973; Ivry, 1997). As a site of procedural memory, the cerebellum has been predicted to operate as a *learning* machine (Marr, 1969; Ito, 2006). It receives the motor commands from cerebral cortex and, through internal memory of movement inverse dynamics, it is able to elaborate a *prediction* of sensory consequences of motor acts. The sensory prediction is then compared to the sensory feedback to produce a sensory discrepancy signal (Blakemore et al., 2001; Ivry et al., 2002; Ivry and Spencer, 2004). This triad—namely *learning, timing* and *prediction*—emerges as a crucial determinant in adaptive behavior under cerebellar control (De Zeeuw et al., 2011; D’Angelo and Casali, 2012).

Afferent signals are conveyed to the cerebellum through two excitatory pathways composed of the MFs and the CFs. Both these fiber systems send collaterals to the DCN before entering the cerebellar cortex. The MFs contact GrC dendrites in the granular layer of the cerebellar cortex. The GrCs axons generate the PFs, that ascend to the molecular layer and relay the signals onto PCs dendritic arbors. Moreover, PCs directly receive the CFs input and in turn inhibit the DCN. Inhibition in the cerebellar cortex is provided by GoCs interneurons in the granular layer, and stellate (SCs) and basket cells (BCs) in the molecular layer. The DCN cells are inhibited by PCs axons and activated by MF and CF collaterals (Fredette and Mugnaini, 1991; Teune et al., 1995, 2000; Medina et al., 2002). While the PCs represent the only output of the cerebellar cortex, the DCN neurons integrate the PC inhibitory input with the excitatory inputs carried by CFs and MFs collaterals and provide the sole output of the cerebellum.

The regular architecture of the cerebellum has inspired several theories, aiming at understanding how the cerebellum processes incoming information and performs timing and learning functions. According to the *motor-learning theory* (Marr, 1969; Albus, 1971), the property of learning motor skills relies on the cerebellar cortex ability to store stimulus-response associations, by linking inputs with the appropriate motor output. The theory implied that only PF-PC synapses may be modified by experience and that the CF acting as a teacher signal calibrates the PC responsiveness and thus leads the encoding of stimulus-responses associations. The motor-learning theory in the Marr’s version implies that, when MFs carry inappropriate information, the PF-PC synapse should be silenced by the olivary input (the opposite would occur according to Albus’ version). The hypothetical plasticity of PF synapses postulated by the Motor Learning Theory was observed *in vivo* as a persistent attenuation of PF-PC transmission (PF-PC long term depression, LTD) produced when PF and CF inputs are stimulated together at low frequency (Ito, 1972, 1989). Miles and Lisberger proposed an alternative model (valid at least for the VOR), in which motor learning is achieved through synaptic plasticity at a different site. The instructive signal conveyed by the PC to the vestibular nuclei triggers a change in synaptic efficacy in the connection between MF collaterals and vestibular nuclei (Miles and Lisberger, 1981).

Experimental data provided support for and against each of the two hypotheses, indicating that the explanation of cerebellar motor learning is likely to involve a more complex picture than plasticity at a single synapse. The cellular basis of cerebellar motor learning is generally assumed to be mediated by long-term modifications in the strength of synaptic transmission (for review see Martin et al., 2000). However, the information storage may also involve activity dependent changes in neuronal intrinsic excitability (Armano et al., 2000; Hansel et al., 2001; Zhang and Linden, 2003; Frick and Johnston, 2005; Mozzachiodi and Byrne, 2010).

Different forms of synaptic and non-synaptic plasticity have been described in excitatory and inhibitory neurons of the granular layer, the molecular layer and the DCN (Hansel et al., 2001; Boyden et al., 2004; Gao et al., 2012). Thus, it is likely that cerebellar learning emerges as an integrated process involving various synaptic sites that elaborate, over different time courses, different components of learning (Medina and Mauk, 2000; Medina et al., 2000; van Alphen and De Zeeuw, 2002; Jörntell and Ekerot, 2003; Yang and Lisberger, 2013). However, how remodeling of synaptic weights generates the complex properties of cerebellar learning remains to be understood.

Mathematical models (Mauk and Donegan, 1997; Medina and Mauk, 1999, 2000; Medina et al., 2000, 2001; Ohyama et al., 2003b; Mauk and Buonomano, 2004; Lepora et al., 2010) incorporating more and more details on synaptic connectivity and plasticity at different network sites, may help determining the impact of the different sites of plasticity on cerebellar learning (Shadmehr et al., 2010). These computational approaches have generated several hypotheses, many of which require validation through experimental assessment. Specific tests can be performed either by using mutant mice with alterations in specific plasticity mechanisms (Gao et al., 2012) or by embedding a cerebellar model with multiple learning rules into the control loop of a robotic simulator (Garrido et al., 2013a; Casellato et al., 2014, 2015).

## Excitatory and Inhibitory Plasticity in the Granular Layer

*In vitro*, LTP and LTD at the MF-GrC synapse are associated with changes of GrCs intrinsic excitability (D’Angelo et al., 1999; Armano et al., 2000; Sola et al., 2004; Gall et al., 2005; Nieus et al., 2006; D’Errico et al., 2009). *In vivo*, LTP and LTD can be induced in the granular layer by facial tactile stimulation and intra-cerebellar electrical stimulation (Roggeri et al., 2008). In mathematical models using reconvolution algorithms of granular layer local field potentials, the synaptic and non-synaptic changes reported *in vitro* turned out to be necessary and sufficient to explain those observed *in vivo* (Diwakar et al., 2011). Information on potential changes in the inhibitory circuit are poor at the moment, but they may be synergistic or antagonistic with respect to those at the MF-GrC relay and regulate information transfer through the granular layer (Arleo et al., 2010; Garrido et al., 2013b).

### Plasticity at the MF-GrC Synapse

MF-GrC LTP induction is driven by the coactivation of NMDA receptors (NMDARs) and metabotropic glutamate receptors (mGluRs) (Rossi et al., 1996; D’Angelo et al., 1999; Maffei et al., 2002). The NMDARs are the main source of Ca^2+^ influx that drives synaptic plasticity induction at the MF-GrC relay, while mGluRs represent an amplifying mechanism acting through the IP3 pathway (Finch et al., 1991; Irving et al., 1992a). Voltage-Dependent Calcium Channels (VDCCs) activation, following membrane depolarization and repetitive spike discharges, may also favor MF-GrC LTP (Armano et al., 2000). The intracellular Ca^2+^ signals may be remarkably protracted and amplified by Ca^2+^-induced Ca^2+^ release (CICR) mechanisms (Irving et al., 1992a,b; Simpson et al., 1996).

Knowing the mechanisms underlying LTP/LTD balance is fundamental to understand how the information is processed and retransmitted by the granular layer. Several intrinsic and extrinsic factors could regulate bidirectional plasticity (for review see D’Angelo, 2014).

First, the patterns of MFs stimulation determine LTP-LTD balance. According to the BCM learning rule (Bienenstock et al., 1982), long-term synaptic plasticity is correlated with the duration of stimulus trains through postsynaptic Ca^2+^ regulation (Gall et al., 2005). Long and repeated MF bursts induce a relatively large Ca^2+^ influx that drives LTP. Instead, LTD is induced by short isolated burst stimulation that causes relatively small Ca^2+^ changes (Gall et al., 2005). Similarly, bidirectional plasticity is influenced by MF stimulation frequencies (D’Errico et al., 2009).

Secondly, nitric oxide (NO) may orchestrate the LTP/LTD balance. High frequency MF stimulation generates a significant NMDAR-dependent and NOS-dependent release of NO in the granular layer (Maffei et al., 2003). As a retrograde messenger, NO regulates the presynaptic release probability, thus driving LTP expression (Maffei et al., 2002). NO release inhibition shifts the balance toward LTD, suggesting that NO is critical for determining plasticity orientation (Maffei et al., 2003).

Thirdly, gating by neuromodulators has been proposed to control LTP and LTD induction at the MF-GrC relay (Schweighofer et al., 2001, 2004). Indeed, it has recently been shown that the cholinergic system enhances MF-GrC LTP through α7-nAChRs activation by shifting the Ca^2+^-plasticity relationship. In this way, in the presence of nicotine a short MF burst that normally induces MF-GrC LTD, is able to induce LTP, both in acute brain slices and *in vivo* (Prestori et al., 2013). The cholinergic facilitation of LTP induction could be critical for controlling adaptive behaviors like VOR (Schweighofer et al., 2001, 2004; Prestori et al., 2013).

Finally, the combination of synaptic response (excitatory post-synaptic potential, EPSP) and spikes has itself a role in determining plasticity, giving rise to the so-called spike-timing-dependent-plasticity (STDP; Song et al., 2000). Preliminary evidence suggests that STDP could indeed exist at the MF-GrC relay, although the underlying mechanisms remain to be clarified (Sgritta et al., 2014).

### Plasticity in the GoC Inhibitory Circuit

Plasticity in the GoC inhibitory circuit may regulate information transfer at the MF-GrC synapse. Following protracted high frequency activation of the MF bundle (typically a theta burst stimulation, TBS) the long-term synaptic plasticity in the granular layer shows a specific spatial organization (Mapelli and D’Angelo, 2007). In particular, LTP and LTD are organized in center-surround structures: more active centers that tend to generate LTP, and less active surrounds that preferentially generate LTD. The sign of plasticity depends on the excitatory/inhibitory balance and therefore it is sensitive to the inhibitory circuit activity. Therefore, GoCs activity may modulate the center-surround organization of signal transmission and bidirectional plasticity at the MF-GrC relay (Mapelli and D’Angelo, 2007; Mapelli et al., 2010, 2014). Although a form of long-term plasticity in the inhibitory GoC-GrC connection has not been described, a recent model suggests that it could represent a potent regulatory mechanism for MF-GrC plasticity (Garrido et al., 2013b). A form of LTD at PF-GoC synapse, following high-frequency burst stimulation of PFs (Robberechts et al., 2010) and long term enhancement of spontaneous GoC firing rate after hyperpolarization (Hull et al., 2013) have been reported (with the first potentially being synergistic and the second homeostatic with respect to the MF-GrC pathway). Also forms of adaptation and long-lasting regulation at the GoC-GrC synapse have been described (Rossi et al., 2006; Mapelli et al., 2009; Brandalise et al., 2012), operating a disinhibition of GrCs in case of high GoCs activation rates, in that being presumably synergistic in our case. However, plasticity at this level remains to be fully investigated. A control of GoC inhibitory activity could also come from GoC-GoC inhibitory synapses (Hull and Regehr, 2012) and gap-junctions (Vervaeke et al., 2010), although the potential impact of these mechanisms on plasticity in the inhibitory circuit is unclear.

### Plasticity of GrC Intrinsic Excitability

High frequency activation of the MF-GrC relay (either through a TBS or a high frequency/protracted stimulation) has been shown to induce long-term modifications of GrC intrinsic excitability, along with the LTP of synaptic efficacy. In particular, high frequency stimulation (HFS) is able to determine a long-lasting increase in neuronal responsiveness, increasing the GrC input resistance and reducing the spike threshold. This form of plasticity had been described for the first time in the hippocampus by Bliss and Lomo in 1973 (Bliss and Lomo, 1973). At the MF-GrC synapse, protracted high-frequency stimulations, weaker than the TBS, determine the increase of the intrinsic excitability, leaving unaltered the postsynaptic response amplitude. As for the synaptic LTP, the plasticity of GrC intrinsic excitability is dependent on NMDARs activation (Armano et al., 2000). Indeed, a previous work assessed the role of the NMDA current in enhancing synaptic depolarization and GrC output firing, in particular in response to HFSs (D’Angelo and Rossi, 1998). The mechanism involved in the NMDAR-dependence probably relies on the calcium ions influx through these receptors (and the consequent activation of calcium-dependent intracellular pathways), rather than on the depolarization consequent to NMDARs opening (Armano et al., 2000). The spike threshold reduction is probably related to a modification of the persistent sodium current and potassium currents (Nieus et al., 2006). The potentiation of GrC responsiveness, and the consequent increase in the number of emitted spikes, reasonably facilitates the development of synaptic LTP. Plasticity of GrC responsiveness could have an additional compensatory role, by restoring granular layer level of excitability in case of weak synaptic excitation (Frégnac, 1998; Armano et al., 2000). Both these mechanisms (synaptic long-term plasticity and plasticity of intrinsic excitability) cooperate in determining the granular layer processing of MF input. Indeed, GrC electrotonic compactness (Silver et al., 1992; D’Angelo et al., 1993) presumably determines that a change in intrinsic excitability would affect neuronal responsiveness as a whole, including synaptic efficacy. Synaptic inhibition mediated by GoCs reasonably affects GrC excitability, through tonic and phasic mechanisms (Armano et al., 2000; D’Angelo et al., 2005). Indeed, GABAergic inhibition modulates GrC excitability in different ways ((Brickley et al., 1996; Rossi et al., 2006); for review (Mapelli et al., 2014)).

## Excitatory and Inhibitory Plasticity in the Molecular Layer

The Marr-Albus-Ito hypothesis of cerebellar motor learning implies that the PF input to PCs is the only site of learning in the cerebellar network. However, multiple sites of synaptic plasticity in the molecular layer have been described (Hansel et al., 2001; Boyden et al., 2004; Coesmans et al., 2004; Ito, 2006). The picture emerging from the latest evidences shows that several forms of synaptic plasticity, not only the classical PF-PCs LTD, appear to be involved in cerebellar learning. Here we summarize the principal features of molecular layer plasticity (for a detailed review see Gao et al., 2012; D’Angelo, 2014).

### Plasticity at the PF-PC Synapse

Different forms of LTP and LTD, either entirely postsynaptically or presynaptically expressed, have been observed at the PF-PC relay. Thus, four different forms of plasticity may be described: a postsynaptic LTD, a postsynaptic LTP, a presynaptic LTP and a presynaptic LTD.

*Postsynaptic PF-PC LTD* is induced by paired PF and CF stimulations and involves complex signal transduction pathways. The activation of AMPARs and mGluRs following PF stimulations induces, through different mechanisms, a postsynaptic Ca^2+^ transient that, over a certain threshold, may activate protein kinase C (PKC; Hartell, 2002). Active PKC phosphorylates the AMPARs at the PC terminals and drives their desensitization and internalization, resulting in LTD of PF-PC relays (Wang and Linden, 2000; Xia et al., 2000). CF stimulations contribute to generate large widespread Ca^2+^ transients, through AMPARs, NMDARs, and VGCCs activation (Konnerth et al., 1992; Piochon et al., 2010). In particular, postsynaptic CF-PC NMDARs are necessary for LTD (but not for LTP) at the PF-PC synapse, when PF activation is paired with CF activation (Piochon et al., 2010). CaMKIV (Boyden et al., 2006) and α/βCaMKII (Hansel et al., 2006; van Woerden et al., 2009) are necessary both for PF-PC LTD and for motor learning. Nevertheless, this form of LTD does not strictly require CF activity and may be induced through PF stimulation alone (Ohtsuki et al., 2009). Other mechanisms could amplify local Ca^2+^ signals and allow LTD induction, as somatic depolarization (Linden et al., 1991) or strong PFs activation (Hartell, 1996; Eilers et al., 1997). Indeed, the simultaneous activation of several PFs stimulated at 1 Hz, at relatively high stimulus intensity, may generate postsynaptic Ca^2+^ transients that remain confined to spines (Midtgaard et al., 1993; Denk et al., 1995), reaching the levels for the LTD induction (Hartell, 1996). However, when the CFs are stimulated a lower stimulus strength is sufficient for LTD induction (Han et al., 2007). Therefore, although it is clear that CF activity facilitates PF-PC LTD, CF involvement is not strictly required (Ohtsuki et al., 2009). Additionally, intense PF stimulations (as brief burst of 2–5 pulses at 10–50 Hz for 1–2 min) may induce heterosynaptic LTD (Marcaggi and Attwell, 2007), in which the LTD may spread to PF synapses tens of microns away from the original site, through second messengers such as NO (Reynolds and Hartell, 2000; Wang et al., 2000) and arachidonic acid (Reynolds and Hartell, 2001). The NO pathway is necessary for the heterosynaptic LTD induction (Crepel and Jaillard, 1990; Shibuki and Okada, 1991; Daniel et al., 1993) and provides another important mechanism involved in plasticity induction at the PF-PC synapses. Indeed, the NO produced by PFs (Southam et al., 1992; Kimura et al., 1998) or by MLIs (Carter and Regehr, 2000) activates a NO-dependent form of guanylate cyclase (GC) in PCs, thus activating the cGMP/PKG pathway, whose effect is to prevent the dephosphorilation of AMPARs by blocking the PP2/PP1/PP2B cascade and therefore unblocking PKC (Lev-Ram et al., 1995, 1997; Linden et al., 1995; Gao et al., 2012).

*Postsynaptic PF-PC LTP* is induced by single pulses PF stimulation at 1 Hz for 5 min, driving GluR2 AMPARs subunit insertion in the spine membrane, through a mechanism dependent on the activation of the PKA, PKC and CAMKII pathways (Lev-Ram et al., 2002; Coesmans et al., 2004; Belmeguenai and Hansel, 2005; Kakegawa and Yuzaki, 2005; van Woerden et al., 2009). The use of selective mutant mice can help investigating the mechanisms underlying these forms of plasticity and their role *in vivo*. In particular, the L7-PP2B mice, in which the PP2B was deleted only in cerebellar PCs, allowed to determine that this molecule is necessary for PF-PC LTP and for correct VOR and eye-blink conditioning (Schonewille et al., 2010). Postsynaptic LTP and LTD are mutually reversible, modulating the AMPARs desensitization and membrane expression (Lev-Ram et al., 2003; Coesmans et al., 2004). The sign of plasticity is determined by several factors, depending on the different induction mechanisms, the NO pathway and the postsynaptic Ca^2+^ transients. In general, stimulation patterns that generate a relatively low Ca^2+^ influx drive LTP while relatively high Ca^2+^ transients are associated with LTD (Coesmans et al., 2004). This is an opposite scenario of that predicted by the “BCM rule”, in which lower and higher Ca^2+^ transients are associated with the induction of LTD and LTP respectively (Bienenstock et al., 1982). This property of PF-PC plasticity may have profound impact on cerebellar information processing.

*Presynaptic PF-PC LTP* may be induced by low-frequency (2–8 Hz) PF stimulations (Sakurai, 1987; Crepel and Jaillard, 1990; Hirano, 1991; Shibuki and Okada, 1992), determining an increase of presynaptic Ca^2+^ influx that activates the adenyl cyclase (AC1) pathway. The consequent activation of PKA determines the phophorilation of the vesicle-release related proteins thus increasing neurotransmitter release (Salin et al., 1996; Kimura et al., 1998; Storm et al., 1998; Powell et al., 2004). In addition, NO released by neighboring synapses may regulate the probability of glutamate release and LTP induction in non-activated PF terminals (Hartell, 2002; Qiu and Knöpfel, 2007; Le Guen and De Zeeuw, 2010). On the contrary, the endocannabinoids released after a high frequency bursts, suppress in PF terminals the AC1 pathway, activating the cannabinoid 1 (CB1) receptors, thereby preventing the induction of presynaptic LTP (van Beugen et al., 2006). A cannabinoid-mediated affect at this level has been described as consequent of the activation of the cholinergic system, mediated by muscarinic receptors (Rinaldo and Hansel, 2013).

*Presynaptic PF-PC LTD* also requires the activation of CB1 receptors. This LTD may emerge after a low-frequency stimulation when presynaptic LTP is pharmacologically prevented, providing a mechanism of bidirectional plasticity at the presynaptic site (Qiu and Knöpfel, 2007).

In conclusion, the cholinergic system and endocannabinoid receptors are able to deeply modulate synaptic plasticity at the PF-PC connection, at various levels. Notably, these two system proved able to interact (Rinaldo and Hansel, 2013). As it is true also for other cerebellar regions, cholinergic activation is able to modulate synaptic activity and plasticity, also mediating the release of other neurotransmitters, therefore influencing local neuronal activity (Turner et al., 2011).

### Plasticity at the CF-PC Synapse

The CFs activity may play an important role in regulation of LTP/LTD balance at the PF-PC synapses. The CF stimulation facilitates postsynaptic PF-PC LTD induction by enhancing dendritic Ca^2+^ signals and by releasing the neuropeptide CRF. Moreover, CF activity triggers the release of endocannabinoids from PC dendrites and suppresses the presynaptic PF-PC LTP (Ohtsuki et al., 2009). The high probability of neurotransmitter release at the CF terminals (Dittman and Regehr, 1998) as well as the all-or-none character of CF signaling (Eccles et al., 1966) has induced to consider the CF-PC synapses as “unmodified” synapses. However, low-frequency (5 Hz, 30 s) CF stimulation may induce LTD of PC responses (Hansel and Linden, 2000; Carta et al., 2006). The CF-LTD is postsynaptically induced and expressed (Shen et al., 2002) and it is associated with an alteration in the complex spike waveform (Hansel and Linden, 2000), a reduction in the complex spike afterhyperpolarization (Schmolesky et al., 2005), and a depression of CF-evoked dendritic Ca^2+^ transient (Weber et al., 2003). The CF-LTD has a significant effect on the probability of induction of postsynaptic LTD and LTP at PF-PC synapses (Coesmans et al., 2004). The reduction in complex spike-associated Ca^2+^ transients following the CF-LTD is sufficiently strong to reverse the polarity of postsynaptic plasticity at the PF-PC relay. Indeed, when CF-LTD is induced first, subsequent application of PF-PC LTD induction protocol results in LTP (Coesmans et al., 2004). A form of CF-PC LTP has been described during development in mice (around 4–11 postnatal days) (Bosman et al., 2008; Ohtsuki and Hirano, 2008). This LTP requires large CF inputs and is dependent on postsynaptic Ca^2+^ increase although being independent on NMDARs activation (Bosman et al., 2008). Since CF innervations on PC shows a 1:1 ratio in adult animals (while more CF impinge on the same PC during development), the CF-PC LTP observed in newborn mice could help strengthen one CF connection, while determining the pruning of the others (Bosman et al., 2008; Ohtsuki and Hirano, 2008).

### Plasticity in the MLI Inhibitory Circuit

PF-MLI synapses and MLI-PC synapses are both sites of plasticity. Different forms of long-term plasticity have been described in PF-MLI relays: a postsynaptic LTD, a postsynaptic LTP and a presynaptic LTP.

Postsynaptic PF-MLI LTD can be induced by sustained PFs stimulations (repeated sequences of 4 × 25 stimuli at 30 Hz) and requires the activation of Ca^2+^-permeable AMPARs, mGlur1Rs and CB1Rs. The postsynaptic Ca^2+^ influx that drives LTD induction is confined at activated synapses (Soler-Llavina and Sabatini, 2006). Moreover, this synapse-specific plasticity drives the membrane expression of GluR2-containg Ca^2+^-impermeable AMPARs, thus providing a self-limiting mechanism (Liu and Cull-Candy, 2000; Sun and June Liu, 2007). PFs stimulation paired with SCs depolarization, which could follow CFs activations (Szapiro and Barbour, 2007), can induce a postsynaptic PF-MLI LTP (Rancillac and Crépel, 2004). This LTP depends on NO and/or cAMP (Rancillac and Crépel, 2004). *In vivo* PF-MLI LTP may be induced by simultaneous activation of PFs and CFs inputs, resulting in long-lasting increases in receptive fields of MLIs (Jörntell and Ekerot, 2002).

Presynaptic LTP at PF-MLI synapses has been described after PF stimulations (at 8 Hz for 30 s (Bender et al., 2009)) and provides a positive feedback mechanism. Indeed, GABA released from MLI diffuses in the extracellular space and activates GABAARs at nearby PF terminals. GABAARs activation leads to an increase in PF release probability and an increase of the excitability of the axon and soma/initial segment, potentiating synaptic transmission onto MLI (Pugh and Jahr, 2011).

CF activation can induce a long-lasting potentiation of PCs spontaneous and evoked inhibitory post-synaptic currents (IPSCs), a phenomenon that is called *rebound potentiation* (Kano et al., 1992). This form of MLI-PCs LTP requires the increase of intracellular Ca^2+^ concentration and is caused by the upregulation of GABAAR activity on PCs (Kano et al., 1996; Hashimoto et al., 2001; Kawaguchi and Hirano, 2007).

There are therefore several mechanisms that could come into play to make synaptic plasticity in the MLI inhibitory circuit either synergistic or antagonistic with respect to plastic changes occurring at the PF-PC synapse.

### Plasticity of PC Intrinsic Excitability

PC excitability may be enhanced by somatic current injection or the PFs stimulation protocols that induce PF-LTP (Belmeguenai et al., 2010). The PCs intrinsic plasticity shares with LTP the activation of protein phosphatises 1, 2A and 2B for the induction (Belmeguenai et al., 2010). It also requires PKA and casein kinase 2 (CK2) activity and it is mediated by the downregulation of different K^+^ channel-mediated conductances, such as A-type K^+^ channels and probably Ca^2+^-activated K^+^ currents (Schreurs et al., 1998). PC intrinsic plasticity, resulting in enhanced spine Ca^2+^ signaling, lowers the probability of subsequent LTP induction. Thus, intrinsic PC plasticity follows LTP of active PF synapses and reduces the probability of subsequent LTP at weaker, non-potentiated synapses.

## Excitatory and Inhibitory Plasticity in DCN

The DCN (as well as the vestibular nuclei, VN) are strategically located within the cerebellar circuitry, in a position ideal to integrate the information coming from brain stem, inferior olive (IO) and spinal cord with the PCs output coming from the cerebellar cortical loop, and provide the sole output of the cerebellum. Experimental investigations using pharmacological tools and focal lesions have revealed that the DCN play an important role in associative learning, such as in eyelid conditioning or VOR adaptation (Lavond et al., 1985; Steinmetz et al., 1992). Evidence that these forms of cerebellar motor learning induce plasticity in DCN and VN (Lisberger, 1994; du Lac et al., 1995; Kleim et al., 2002; Ohyama et al., 2006) suggested that memory storage was not limited to the cerebellar cortex. Long-term modifications in synaptic strength have been described in the inhibitory synapses between PCs and DCN neurons and in the excitatory synapses between MFs and DCN. In addition, persistent changes of the intrinsic excitability have been observed in DCN neurons. Recently, it has been suggested that PC-DCN and MF-DCN synapses are plastic on a slow time scale and store persistent memory. Conversely, plasticity in cerebellar cortex could operate on a shorter time scale, storing transient memory that could then be transferred downstream and consolidated through DCN plasticity in slow phases of learning (Medina and Mauk, 1999; Medina et al., 2000; Masuda and Amari, 2008).

### Plasticity at the MF-DCN Synapse (Excitatory)

DCN neurons show a robust post-inhibitory rebound spike burst after stimulation of inhibitory PCs synapses (Gardette et al., 1985a,b; Aizenman and Linden, 1999). This rebound hallmark induced by PC activity drives the plastic changes of the MF-DCN glutamatergic synapse. A MFs high-frequency burst that precedes a DCN post-inhibitory rebound depolarization induces a synapse-specific MF-DCN LTP (Pugh and Raman, 2006). This LTP induction protocol mimics the predicted time course of excitation and inhibition during delay eyelid conditioning. The MFs convey the conditioned stimulus, while the unconditioned stimulus is carried by the CFs. The DCN neurons receive excitation directly from the MFs collaterals, followed by the indirect inhibition via the GrC-PC-DCN circuit (Mauk et al., 1986; Hesslow et al., 1999). The PCs respond to the unconditioned stimulus with a complex spike, followed by a brief pause that allows the post-inhibitory firing in the nuclei. During cerebellar learning of associative tasks, the acquisition of the conditioned response depends on the correct timing between the MF-mediated excitation and the PC-mediated inhibition that drives excitatory post-synaptic currents (EPSCs) potentiation (Ohyama et al., 2003a). The PCs fire as a response to MF activation, but when the conditioned and unconditioned stimuli are paired and repeated, PCs firing slows down during the final phase of the conditioned stimulus. This would generate a disinhibition in DCN neurons, allowing the generation of the excitatory response that elicits a blink (Jirenhed et al., 2007). LTP cannot be induced when the timing of synaptic excitation and hyperpolarization is modified. With longer intervals between excitation and inhibition, or with a reverse sequence, EPSCs tend to depress (Pugh and Raman, 2008).

MF-DCN LTP depends on both NMDAR and low-voltage-activated Ca^2+^ channels, activated respectively by synaptic excitation and inhibition (Pugh and Raman, 2006). DCN are spontaneously active neurons and express NR2D subunit-containing NMDARs, generating channels weakly blocked by Mg^2+^ (Akazawa et al., 1994). Therefore, unlike other brain regions, the MF-DCN LTP is not consequent to the coincidence detection of signals that generates a suprathreshold increase in the intracellular Ca^2+^ level. MF-DCN plasticity rather depends on the timing of two different signals that act independently to activate distinct intracellular signaling pathways. This mechanism may be adequate to encode temporal information that is required for non-Hebbian and adaptive plasticity during associative learning tasks (Medina and Mauk, 1999; Pugh and Raman, 2009). The excitation drives the Ca^2+^ influx in individual synapses, with NMDARs providing the priming signal. The inhibition generates a global signal that triggers LTP induction only in the primed synapses (Pugh and Raman, 2008). Multiple signaling cascades may be activated by priming and trigger signals. The Ca^2+^ influx through NMDARs activates the calcium-dependent phosphatase calcineurin, while the Ca^2+^ influx through the high-voltage-activated Ca^2+^ channels activates the calmodulin-dependent protein kinase II (CaMKKII). At the same time, the potentiation of the primed synapse is triggered only if the suppressive effect of L-type Ca^2+^ current is reduced by hyperpolarization (Person and Raman, 2010). This provides evidence that synaptic inhibition plays an active role in the induction of MF-DCN LTP.

Moreover, a form of MF-DCN LTD has been reported, which can be induced by MFs high-frequency burst stimulation, either alone or paired with postsynaptic depolarization. Again, a postsynaptic Ca^2+^ transient is needed to the induction of this plasticity, that it is blocked by Ca^2+^ chelators (Zhang and Linden, 2006). MF-DCN LTD is NMDAR independent and requires the activation of the group I metabotropic glutamate receptor 1 (mGluR1) and protein translation (Zhang and Linden, 2006).

### Plasticity at the PC-DCN Synapse (Inhibitory)

LTP of IPSCs in DCN neurons can be induced after HFS at 100 Hz (Ouardouz and Sastry, 2000) of PC axons, while stimulation at lower frequencies (as 10 Hz), induces LTD (Morishita and Sastry, 1996). The PC-DCN tetanus-induced long-term plasticity does not require GABARs activation (Morishita and Sastry, 1996; Ouardouz and Sastry, 2000). LTP and LTD appear to depend on NMDAR activation and on intracellular Ca^2+^ increase, as they are both blocked by NMDAR antagonist APV and/or the calcium chelator BAPTA. Moreover, depolarizing pulses that activate VGCCs in DCN neurons induce LTP when given at 2 Hz or LTD when given at 0.1 Hz (Morishita and Sastry, 1996; Aizenman et al., 1998; Ouardouz and Sastry, 2000). Therefore, a large Ca^2+^ influx through NMDAR or L-Type Ca^2+^ channels drives LTP of IPSCs, while LTD is induced by moderate Ca^2+^ increases. The plasticity induced by depolarization pulses is weaker than that induced by tetanus (HFS). The Ca^2+^ increase that follows the depolarizing pulses driving a smaller LTP, remains mainly located in the DCN soma and proximal dendrites (Muri and Knöpfel, 1994; Aizenman et al., 1998). In contrast, HFS may act also on CFs and MFs collaterals, whose excitatory synapses are distributed on DCN soma as well as on proximal and distal dendrites (Ikeda and Matsushita, 1973, 1974). The consequent NMDARs activation leads to a larger increase of intracellular calcium, both in the soma and in the dendritic tree, inducing a stronger IPSCs LTP.

PCs activity drives the plasticity of the inhibitory synapses in DCN neurons, but the sign of the bidirectional plasticity strikingly depends on excitatory synapses activation level. Therefore, the activation of MF or CF collaterals influences the induction of LTP (Ouardouz and Sastry, 2000) or LTD (Morishita and Sastry, 1996), by regulating the Ca^2+^ influx through the NMDARs.

### Plasticity of DCN Intrinsic Excitability

High-frequency MF bursts induce potentiation of intrinsic excitability in DCN neurons (Aizenman and Linden, 2000; Zhang et al., 2004). After the EPSP bursts, the input resistance and the number of action potentials evoked by a depolarization pulse increase, while the spike threshold decreases. Also the rebound depolarization that follows a hyperpolarization step is increased. All these mechanisms enhance DCN neurons excitability. Similar to the GrC intrinsic excitability increase following MF-GrC LTP (Armano et al., 2000), the changes in DCN intrinsic excitability depend on NMDARs activation and require an increase in intracellular Ca^2+^ concentration. MF stimulation may also drive LTP in DCN (Pugh and Raman, 2006), suggesting that potentiation of intrinsic excitability coexists with potentiation of synaptic efficacy, again similar to MF-GrCs LTP. The increase in neuronal excitability amplifies synaptic potentiation, enhancing the ability of DCN neurons to respond to MF inputs (Zheng and Raman, 2010). Also PC inhibitory post-synaptic potential (IPSP) bursts can induce a persistent and Ca^2+^-dependent increase of DCN intrinsic excitability (Zhang et al., 2004). The changes in DCN neurons excitability caused by PC firing increase followed by a brief pause, might play an important role in motor learning tasks (Zhang et al., 2004).

## Coordination of Multiple Forms of Excitatory and Inhibitory Plasticity

As described above, the three main cerebellar subcircuits (granular layer, molecular layer and DCN) are all sites of complex forms of plasticity, some occurring at excitatory and some at inhibitory synapses (D’Angelo, 2014). However, despite numerous hypotheses have been formulated, the main question remains: how does plasticity at excitatory and inhibitory synapses interact in controlling cerebellar circuit functioning? There are four general considerations that need to be taken into account in order to answer the questions.

First, the granular and molecular layer subcircuits share a similar inhibitory architecture, with a feed-forward inhibitory loop passing through the local inhibitory interneurons and controlling retransmission through the primary neuron (in addition, the granular layer also has a feed-back inhibitory loop). Moreover, the whole cerebellar cortex acts as a third large feed-forward inhibitory loop controlling retransmission through the DCN (Figure 1).

Secondly, synaptic plasticity is present at both excitatory and inhibitory synapses, distributed at several connections at the granular layer, molecular layer and DCN. These different forms of synaptic plasticity are expected to develop in a coordinated manner following signal inputs to the cerebellum.

Thirdly, in each subcircuit, inhibitory interneurons fine-tune the principal neuron output and the critical issue is whether inhibitory plasticity tends to compensate and rebalance changes (*homeostatic effect*) or rather reinforces and amplifies the effects of excitatory plasticity occurring in the main neuronal pathway (*synergistic effect*).

Finally, plasticity is probably dynamically transferred through the cerebellar circuit synapses into deep structures and possibly also outside the cerebellum, e.g., in the cerebral cortex and brainstem (Koch et al., 2008). Cerebellar plasticity seems therefore unavoidably bound to local circuit dynamics (D’Angelo and De Zeeuw, 2009) and to the extended recurrent networks formed by the cerebellum with extracerebellar areas.

### Insight from Experimental Recordings

In order to provide a key to interpret the role of the various plastic mechanisms reported in the cerebellar circuit (see above), it would be helpful to develop a plausible hypothesis of excitatory and inhibitory plasticity interaction using a prototypical demonstration. It is already known that a sensory stimulus like the TSS (theta-sensory stimulus) delivered to the rat whisker pad is able to induce LTD of granular layer response to MF input *in vivo* (Roggeri et al., 2008). Since this effect is expected on the basis of MF-GrC plasticity rules *in vitro* (Armano et al., 2000; Sola et al., 2004; D’Errico et al., 2009), it can be hypothesized that synaptic plasticity at the inhibitory GoC connections does not counterbalance MF-GrC LTD. Therefore, plasticity in the Golgi cell loop may be synergistic with that developed at the MF-GrC relay and may effectively increase GrC inhibition. As a consequence, PFs would convey a decreased level of excitation to the molecular layer. Given the inverse “BCM rule” at the PF-PC connection, a weak PFs activation pattern could lead to PF-PC LTP, as suggested by preliminary data (Ramakrishnan and D’Angelo, 2012). Similarly to the granular layer, the inhibitory feed-forward loop in the molecular layer (PF-MLI-PC) could act synergistically with the PF-PC synapse through the induction of PF-MLI LTD, further boosting PC responses. Little is known about the CFs activation following the TSS, although it is likely that it would considerably affect PF-PC behavior. The consequent increase in PC responsiveness could lead to LTP at the PC-DCN connection, increasing PC inhibition of DCN cells. This, in turn, would favor the onset of post-inhibitory rebound depolarization colliding with MFs high-frequency burst activity conveyed by MFs. Since MF activity precedes DCN post-inhibitory rebound depolarization, MF-DCN LTP would be favored (Pugh and Raman, 2006). This example shows a concatenation of events providing a plausible hypothesis of how excitatory and inhibitory plasticity could act synergistically to modify MF input processing and integration through the cerebellar cortex and DCN. This picture, although deliberately oversimplified, provides a working hypothesis on the events that might develop in cerebellar cortex following patterned inputs on the afferent MF pathway. Clearly, introducing CF inputs and their potential instructive role on PF-PC plasticity is another primary factor that should be considered to reshape the landscape of plasticity and signal transmission through the cerebellar network.

### Insight from Cerebellar Network Models

Cerebellar modeling has traditionally focused on the classical Marr-Albus’ hypothesis of cerebellar learning (Marr, 1969; Albus, 1971), accounting for plasticity only at the PF-PC connection. According to the Marr-Albus’ hypothesis, the cerebellum operates like a perceptron (Albus, 1971). The PF-PC synapses adapt their weights depending on CF activity (assumed to carry error-related signals) and GrC activity (assumed to perform expansion recoding of sensory inputs reaching the cerebellum through the MFs). Although the Marr-Albus’ hypothesis does not account for the numerous forms of cerebellar plasticity and totally ignored any potential role for inhibitory plasticity, it has inspired most cerebellar models elaborated so far. Surprisingly, in these models based on the Marr-Albus’ hypothesis, the cerebellum succeeded in solving different kinds of tasks, including eyelid conditioning (Medina and Mauk, 2000), VOR adaptation (Masuda and Amari, 2008) and object manipulation (Luque et al., 2011) or even multiple tasks together demonstrating generalization (Casellato et al., 2014). The fact is that learning in these tasks was oversimplified and far from biological realism, so that these models provided a proof of principle that the cerebellum requires plasticity to perform sensory-motor control rather than explaining how its internal plasticity mechanisms operate.

Plasticity in the granular layer has long been neglected in computational models. The GrCs have been supposed to sparsify the MFs incoming signals based on a combinatorial principle (Yamazaki and Tanaka, 2007), exploiting their huge number and connectivity. However, some models have proposed that MF-GrCs and GoC-GrC plasticity may improve granular layer sparse coding of MF inputs (Coenen et al., 2001; Schweighofer et al., 2001; Philipona and Coenen, 2004; Rössert et al., 2014). According to these models, Hebbian learning in the MF-GrC synapses, operating in conjunction with anti-Hebbian learning in the GoC-GrC synapses and homeostatic intrinsic plasticity in both GrCs and GoCs, maximizes the information transfer between the MFs and the GrCs, generating a sparse representation of the MF input. Recent models suggested how cerebellar granular-layer coding could take advantage of spike-timing and distributed plasticity (Garrido et al., 2013b; Rössert et al., 2014). Variations in multiple weights distributed among different connections succeeded to regulate the number of GrC spikes and their positioning with millisecond precision in response to MF bursts. The weight at MF-GrC synapses (main transmission pathway) effectively controlled the first-spike delay, as previously shown experimentally (Arleo et al., 2010). Modeling of weight changes at the inhibitory GoC-GrC together with the excitatory MF-GrC connections revealed the key role of inhibition in shaping the timing and precision of GrC firing (Nieus et al., 2014). The weight at MF-GoC synapses (feed-forward inhibitory loop) and PF-GoC synapses (feed-back inhibitory loop) regulated the duration of the excitatory time-window during which the first spike could be emitted. Moreover, the weights at the GoC-GrC synapses (common inhibitory loop) and GoC-GoC (inhibitory interneuron network) weights controlled the intensity and duration of GrC inhibition and the number of emitted spikes. Therefore, plasticity in the inhibitory circuit of the granular layer could effectively shape the spatio-temporal time-windows of PF discharge.

**Figure 2:**
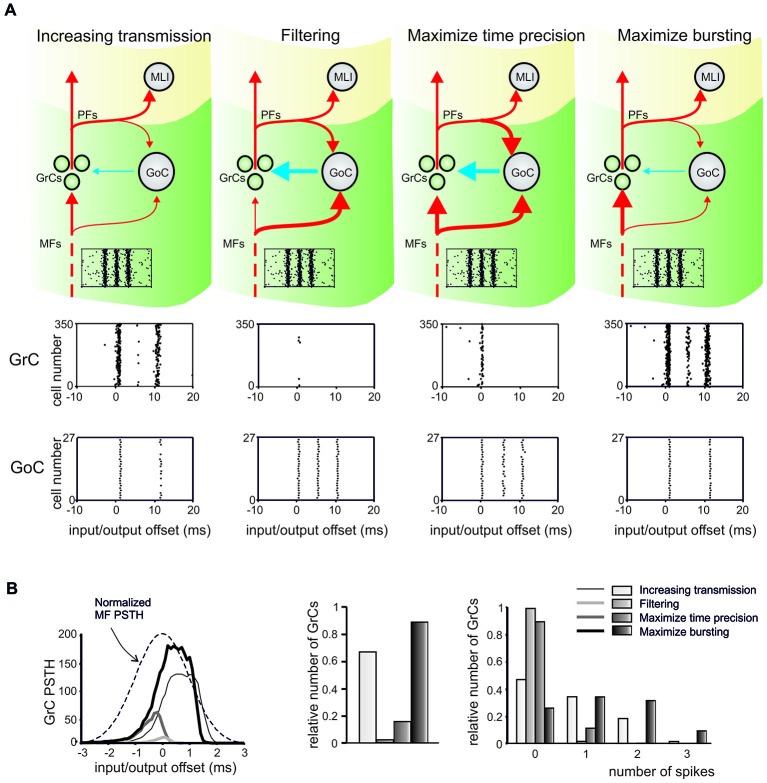
**Integrated regulation of microcircuit functions by synaptic plasticity at excitatory and inhibitory synapses**. This figure shows the effect of results of integrated regulation of microcircuit functions by synaptic plasticity at excitatory and inhibitory synapses in a computational model of the cerebellar granular layer. **(A)** The line thickness in the circuit schemes illustrates the relative synaptic weights for the four different conditions (same colors and circuit elements as in Figure 1) and the raster plots indicate the Mossy fiber (MF) input. Systematic changes in synaptic weights could generate four different effects: (1) *increase transmission*; (2) *signal* *filtering*; (3) *maximize time precision*; and (4) *maximize bursting*. The GrC and GoC firing in response to the MF input burst are shown in the raster plots for each condition. **(B)** The peri-stimulus time histograms (PSTH) show the relative number of GrCs generating spikes in response to the input. The PSTHs change in the four different conditions. The nature of changes is illustrated in the histograms, showing the relative number of GrCs responding to the input pattern with 0, 1, 2, or 3 spikes. *Modified from Garrido et al. (2013b)*.

Distributed plasticity, depending on different combinations of weights at excitatory and inhibitory synapses, proved able to change information flow through the main MF-GrC excitatory pathway favoring different aspects of network processing in turn (Figure 2): (i) increasing transmission (when inhibition on GrCs is depressed); (ii) filtering (in case of inhibition increase and simultaneous LTD at the MF-GrC relay); (iii) maximize time precision (when LTP prevails at all connections in the subcircuit); and (iv) maximize bursting (when inhibition is depressed while the MF-GrC relay potentiates). This model is of particular relevance, since it defines the different functional states that could be achieved by the granular layer circuit in different phases of the learning process. While increasing transmission may be useful to enable the learning process, maximizing timing, filtering or bursting could be the end-point of a specific circuit learning process (Garrido et al., 2013b).

Other models have long hypothesized the role of plasticity in DCN afferent synapses (Medina et al., 2001; Masuda and Amari, 2008; Garrido et al., 2013a; Clopath et al., 2014). These models generally agree that MF-DCN plasticity consolidates the information that has previously been acquired due to the molecular layer plasticity. The separation of learning in two stages (fast learning and consolidation) has been shown to enhance the learning capabilities in eyeblink conditioning (Medina et al., 2001; Monaco et al., 2014), VOR (Masuda and Amari, 2008; Clopath et al., 2014) and complex manipulation tasks (Garrido et al., 2013a). However, this last model has proposed that distributed plasticity in the DCN (including both the MF-DCN and PC-DCN plasticity) could also store gain information, keeping the PCs operating in their optimal firing range and avoiding their saturation. According to this model, while the PF-PC synapses stored information related with the correlation between sensorial state representations (along the sparse GrC activity) and the associated error in the task under development (represented in the CF activity), the DCN afferents could store information about the gain of the task, enhancing the generalization capabilities of the cerebellum. The IO-DCN connection was not considered until a recent computational model proposed that it could act as an internal feed-back loop (Luque et al., 2013), accelerating the convergence of learning without conflicting with the generalization capabilities previously suggested to the MF-DCN and PC-DCN synapses. Moreover, the hypothesized existence of short-term plasticity in that connection could effectively enable/disable this feedback loop based on the error evolution.

**Figure 3:**
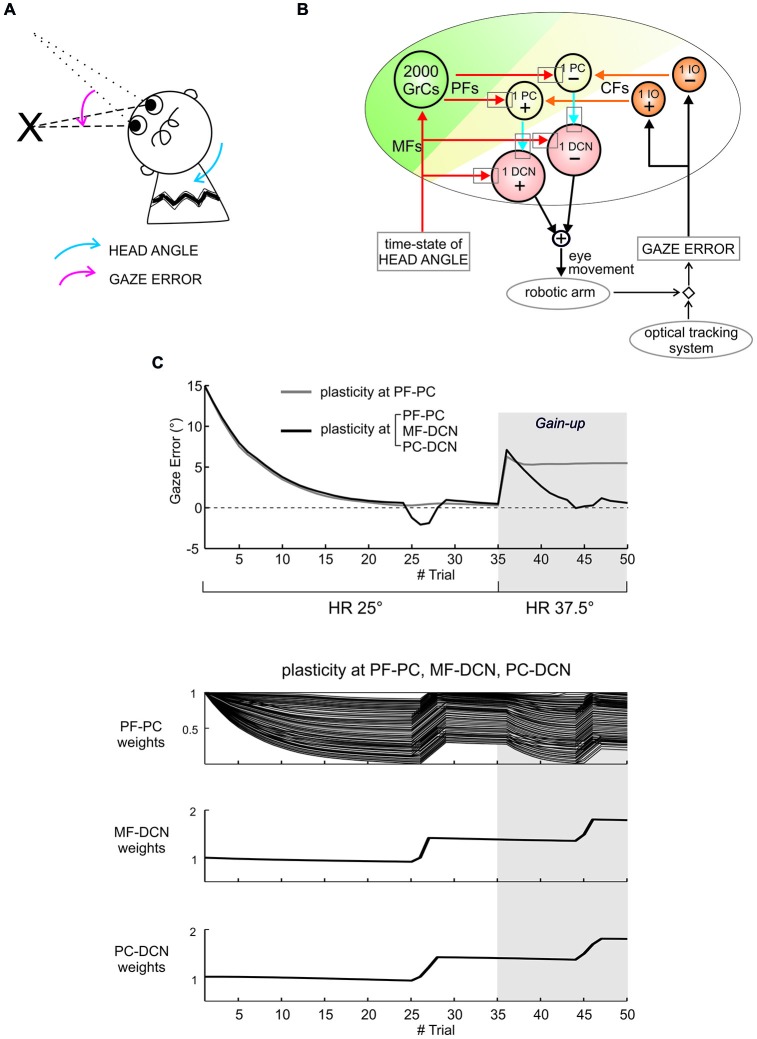
**Distributed cerebellar plasticity in real-robot sensorimotor vestibulo-ocular reflex (VOR) task. (A)** Human-like VOR task: the arrows show the head angle rotation (*blue arrow*) and the angle of gaze error (*pink arrow*). **(B)** Cerebellar model with VOR-specific input and output signals. The plasticity sites are indicated by the *gray rectangles* (all plasticities are bidirectional with LTP and LTD rules defined and calibrated according to experimental observations, see (Casellato et al., 2014, 2015; Luque et al., 2014)). *Red and orange arrows* indicate excitatory connections from mossy fibers (MFs) and CFs, respectively. *Blue arrows* indicate inhibitory connections. The head vestibular stimulus represents the system time-state, decoded by the granular layer. The gaze error is fed into the CF pathway, and the DCN neurons modulate compensatory eye movements. **(C)** Gain-up VOR test: after 35 trials, the head rotation (HR) was increased 1.5 times (from 25° to 37.5°), and imposed for other 15 trials. The curves report the gaze error within each of the total 50 trials, implementing plasticity at a single site (PF-PC connection, *in* *gray*) or at multiple sites (PF-PC, MF-DCN and PC-DCN connections, *in black*). With one or three plasticities, the robot compensated equally well for HR. However, while plasticity at the PF-PC connection alone proved unable to change the gain and to correct for the increased HR, combined plasticities at PF-PC, MF-DCN and PC-DCN were able to rescale the response and adapt to the new HR angle. The three bottom plots show synaptic weights at the end of each trial for the three synapses involved, referring to the case of plasticity at the three connections. Indeed, the transfer from cortical to nuclear sites made the PF-PC synapses ready for further plasticity, making them able to react to perturbations suddenly presented to the system. Modified from Casellato et al. (2015).

These computational models are therefore providing new hypotheses on how inhibitory and excitatory plasticity could integrate to generate the cerebellar output. What is most interesting is that, in *closed-loop robotic simulations*, the multiple forms of long-term synaptic plasticity can effectively enable adaptive motor control with properties—*prediction, timing and learning* (Ivry, 1997; Ivry et al., 2002; Shadmehr et al., 2010)—and temporal dynamics similar to those observed in humans (Casellato et al., 2014, 2015; Luque et al., 2014). The main inhibitory plasticity in these neurorobots was located in the PC-DCN synapse (Figure 3). In these robotic tests, the PF-PC synapse could rapidly learn the contextual information needed to compensate for movement errors. With a slower kinetics, the PC-DCN and MF-DCN synapses were able to store this information in a stable form leaving the PF-PC synapse capable of readapting rapidly. This gave the system a remarkable flexibility preventing PF-PC synaptic weight saturation and allowing its reuse in different tasks, for example when the same muscle district was engaged in different motor tasks and when signal rescaling was needed. Therefore, distributed plasticity seems essential to endow the neuronal circuit with biologically effective properties. Moreover, plasticity at the inhibitory PC-DCN synapse appears to be critical to effectively tune transmission along the large side-loop formed by the cerebellar cortex onto DCN. In future works, more realistic representations of neuronal circuits and learning rules will have to be included into closed-loop robotic systems in order to improve our understanding on how integrated plasticity at inhibitory and excitatory synapses controls functioning of the cerebellar circuit.

## Conclusions

This revisitation of cerebellar network plasticity shows that LTP and LTD at inhibitory synapses, and more in general in the inhibitory loops, are needed to fine tune the activity at crucial neuronal nodes located along the main circuit transmission pathway. The combination of synaptic weight change at excitatory and inhibitory synapses can effectively shape the network activity states. These states could change dynamically in order to enable different phases of the learning process and transfer of plasticity inside and outside the local circuit. Experimental and modeling evidence suggests that, in certain conditions, plasticity at inhibitory and excitatory synapses could have synergistic effects. Therefore, introduction of inhibitory plasticity allows to draw a new picture of cerebellar circuit functioning beyond the original intuition that learning has just to occur through plasticity at the PF-PC synapse. There is now the need for several critical demonstrations to fully understand the integrated role of inhibitory and excitatory plasticity in the cerebellar circuit. First, learning rules at inhibitory and excitatory synapses and their interdependence need to be determined experimentally. This investigation has to take into account the modulatory states and input patterns relevant to control plasticity and may make use of mutant mice with alteration in specific synaptic mechanisms. Secondly, the effective occurrence of LTP and LTD *in vivo* in response to specific stimuli or learning protocols needs to be clarified. Thirdly, network models incorporating realistic learning rules need to be extended in order to simulate plasticity dynamics in the circuit. Finally, closed-loop robotic simulations are needed to determine the effective engagement of network learning mechanisms during complex tasks. In this framework, the cerebellar network is likely to provide a very effective workbench.

## Conflict of Interest Statement

The authors declare that the research was conducted in the absence of any commercial or financial relationships that could be construed as a potential conflict of interest.
